# *HER2* Status in Ovarian Carcinomas: A Multicenter GINECO Study of 320 Patients

**DOI:** 10.1371/journal.pone.0001138

**Published:** 2007-11-07

**Authors:** Marianne Tuefferd, Jérôme Couturier, Frédérique Penault-Llorca, Anne Vincent-Salomon, Philippe Broët, Jean-Paul Guastalla, Djelila Allouache, Martin Combe, Béatrice Weber, Eric Pujade-Lauraine, Sophie Camilleri-Broët

**Affiliations:** 1 JE2492, Université Paris-Sud, IFR69, Villejuif, France; 2 Service de Génétique Oncologique, Institut Curie, Paris, France; 3 Departement de Pathologie, Centre Jean Perrin, Clermont-Ferrand, France; 4 Service de Pathologie, Institut Curie, Paris, France; 5 Centre Léon Bérard, Lyon, France; 6 Centre François Baclesse, Caen, France; 7 Service de Médecine Interne-Oncologie, Centre Hospitalier, Le Mans, France; 8 Centre Alexis Vautrin, Vandœuvre lès Nancy, France; 9 Faculté Paris-Descartes, Assistance Publique - Hôpitaux de Paris (AP-HP), Paris, France; Children's Hospital Boston, United States of America

## Abstract

**Background:**

Despite a typically good response to first-line combination chemotherapy, the prognosis for patients with advanced ovarian cancer remains poor because of acquired chemoresistance. The use of targeted therapies such as trastuzumab may potentially improve outcomes for patients with ovarian cancer. *HER2* overexpression/amplification has been reported in ovarian cancer, but the exact percentage of *HER2*-positive tumors varies widely in the literature. In this study, *HER2* gene status was evaluated in a large, multicentric series of 320 patients with advanced ovarian cancer, including 243 patients enrolled in a multicenter prospective clinical trial of paclitaxel/carboplatin-based chemotherapy.

**Methodology/Principal Findings:**

The *HER2* status of primary tumors and metastases was evaluated by both immunohistochemistry (IHC) and fluorescence *in situ* hybridization (FISH) analysis of paraffin-embedded tissue on conventional slides. The prognostic impact of *HER2* expression was analyzed. *HER2* gene was overexpressed and amplified in 6.6% of analyzed tumors. Despite frequent intratumoral heterogeneity, no statistically significant difference was detected between primary tumors and corresponding metastases.

**Conclusions/Significance:**

Our results show that the decision algorithm usually used in breast cancer (IHC as a screening test, with equivocal results confirmed by FISH) is appropriate in ovarian cancer. In contrast to previous series, HER2-positive status did not influence outcome in the present study, possibly due to the fact that patients in our study received paclitaxel/carboplatin-based chemotherapy. This raises the question of whether HER2 status and paclitaxel sensitively are linked.

## Introduction

Because symptoms are usually absent, 70 to 80% of patients with ovarian cancer will have advanced disease at the time of diagnosis [1]. Despite an initial good response to first-line combination chemotherapy (taxane/platinum), relapses are frequent because of acquired chemoresistance. The use of new targeted therapies that are potentially effective in a subset of patients may be of great value.

*HER2* (human epidermal growth factor receptor-2) proto-oncogene encodes a protein belonging to the EGFR tyrosine kinase receptor family. Overexpression of *HER2* initiates intracellular signaling pathways involved in cell proliferation, differentiation, migration and apoptosis [2]. In breast cancer, *HER2*-positive status is associated with a poor prognosis [3], and also identifies patients who could benefit from anthracycline-based regimens [4].

Trastuzumab (Herceptin®, F. Hoffmann-La Roche, Basel, Switzerland) is a humanized monoclonal antibody that targets the *HER2* extracellular domain and inhibits *HER2-*positive tumor cell proliferation. It is effective alone and in combination with chemotherapy in patients with breast cancer whose tumors express high levels of *HER2* protein. The benefits of trastuzumab have been demonstrated in both metastatic and adjuvant treatment settings [5]–[8].

Accurate evaluation of *HER2* status is essential for optimal patient selection for trastuzumab. Among the numerous methods published, immunohistochemistry (IHC) and fluorescence *in situ* hybridization (FISH) are the most widely used and have high reported concordance [9]–[10]. FISH has been shown to more accurately select patients than IHC, but is more costly and not routinely available. The recommended algorithm for *HER2* determination in breast cancer is to use IHC initially, using a semi-quantitative scoring system followed by FISH for 2+ ambiguous samples [11]–[14].

Observed rates of *HER2* overexpression/amplification in ovarian carcinomas show considerable variation between studies, ranging from 8% to 66% [15]–[33]. Single-agent trastuzumab therapy was associated with a low response rate (7%) in a series of heavily pretreated patients with ovarian cancer [22], but the efficacy of trastuzumab in combination with chemotherapy has not been tested in this setting.

The aim of the current study was to determine the proportion of patients with advanced ovarian cancer whose tumors were *HER2*-positive. This was assessed using conventional slides from both primary tumors and metastases and applying reference *HER2* screening algorithm for breast tumors, to discriminate equivocal and heterogeneous staining.

## Methods

### Participants

Three hundred and twenty patients with advanced primary ovarian carcinomas (International Federation of Gynecology and Obstetrics [FIGO] stage Ic–IV) or primary peritoneal carcinomas were included. Of these, 243 (75.9%) had been enrolled in a larger phase III GERCOR-AGO-OVAR-9 randomized trial of first-line paclitaxel/carboplatin/gemcitabine (TCG) chemotherapy, conducted in 58 centers between July 2002 and April 2004 [ClinicalTrials.gov Identifier: NCT00052468]. The clinical characteristics of patients include in the TCG trial are presented in Table 1. The 77 remaining patients (56 centers) included in our analysis demonstrated paclitaxel/carboplatin chemoresistance (defined as progression during therapy or relapse within 6 months after completing therapy).

**Table 1:** Characteristics of 243 patients included in the phase III TCG trial

Characteristics	N (%)
Age, years	
Median	58
Range	25–77
WHO performance status	
0	99 (40.8)
1	113 (46.5)
2	29 (11.9)
Missing values	2 (0.8)
FIGO stage	
I	11 (4.5)
II	27 (11.2)
III	168 (69.1)
IV	36 (14.8)
Missing values	1 (0.4)
Grade	
Well differentiated (1–2)	97 (39.9)
Poorly differentiated (3–4)	143 (58.9)
Missing values	3 (1.2)
Histological type	
Serous	164 (67.5)
Others	77 (31.7)
Missing values	2 (0.8)
Ascites	
No	97 (39.9)
Yes	109 (44.9)
Missing values	37 (15.2)
Residual tumour after first laparotomy	
Residual disease≤1cm	115 (47.3)
Residual disease >1cm	128 (52.7)

WHO: World Health Organization; FIGO: International Federation of Gynecology and Obstetrics; N: number.

### Ethics

The study was approved by the local ethical committee (CCPPRB number: 02780) and all individual patients gave written informed consent for biological studies.

### *HER2* determination by IHC

Formalin-fixed and paraffin-embedded tumors from primary surgery were obtained retrospectively. Because of previously reported heterogeneity in *HER2* expression in ovarian cancer [24], in the present study we chose to analyze four blocks containing tumor: two blocks of primary tumors and two blocks of chemo-naïve metastases/peritoneal dissemination (available from 206 patients). Most of the metastases analyzed were peritoneal dissemination, whereas few corresponded to lymph node sections.

Following deparaffinization and rehydration, the 4 µm sections were microwave pretreated in pH 6 citrate buffer. Primary antibody (CB-11, Novocastra, Newcastle-upon-Tyne, UK) diluted 1/800 was incubated for 2 hours. Staining was achieved using a streptavidin-biotin-peroxidase kit (Abcys, Biospa, Milano, Italy) including 30-minute incubation for each step. Nuclei were counterstained with haematoxylin. *HER2* positivity was assessed using Ellis and Wolff recommendations [14],[25]. A score of 1+ was defined as barely perceptible membrane staining in more than 10% of cells, a score of 2+ was defined as weak-to-moderate complete membrane with staining present in more than 10% of tumor cells, and a score of 3+ was defined as strong complete membrane staining in more than 10% of tumor cells. We classified 2+ as equivocal and 3+ as positive. Cytoplasmic staining was considered to be non-specific. For each case, an external control containing two FISH-positive (2+/3+) breast cancer samples was used.

### *HER2* determination by FISH

All samples of 2+/3+ *HER2* protein expression and 24 samples with 0/1+ staining score were evaluated by FISH, performed on a single block in one of the following laboratories, according to local instutitional procedures: Institut Curie, Paris (PathVysion *HER2* kit; Abbott-Vysis, Desplaines, IL); Centre Jean Perrin, Clermont-Ferrand (PathVysion *HER2* kit; Vysis, IL); Hôtel-Dieu, Paris, (*HER2* kit, DakoCytomation, Glostrup, Denmark). Instructions from the test kit manufacturers were followed, with slight modifications.

Four µm deparaffinized sections were incubated in pretreatment buffer at 95°C for 15 minutes, then in proteolytic solution at 37°C for 5 minutes. Co-denaturation of the probe and DNA of the tissue section was achieved by incubation at 82°C for 5 minutes using a HyBrite device (Abbott-Vysis) ; this was followed by a 15-hour hybridization at 37°C. Post-hybridization washes were performed according to the respective protocols. Slides were mounted in DAPI/antifade and viewed with a fluorescent microscope. Sixty nuclei in several areas of the section were analyzed, and three representative images per case were captured. Tumors were classified as amplified if they showed a mean of ≥8 *HER2* signals, or a *HER2*/centromere 17 ratio >2.2 in samples of fewer than 8 *HER2* signals.

### Statistics

The study was designed to evaluate *HER2* status in a population of patients with advanced ovarian cancer. *HER2* status in primary tumors and metastases was compared using kappa testing. The relationship between clinical characteristics of the 243 patients included in the TCG trial and *HER2* status was compared using chi-square testing. Overall survival (OS) was calculated from the date of inclusion to death and progression-free survival (PFS) was calculated from the date of inclusion until progression or last follow-up examination. Progression was defined as a 20% increase in the diameter of all measured lesions, appearance of new lesions and/or doubling from baseline of CA125 tumor marker concentration. OS and PFS curves were derived from Kaplan-Meier estimates. A univariate Cox model analysis was performed to estimate and test the prognostic influence of clinical and biological variables. In a multivariate analysis, the Cox proportional hazard regression model was applied to determine the influence of these variables on outcome, adjusted for other prognostic factors. Hazard ratios (HR) and 95% confidence intervals (CI) were determined. The influence of *HER2* status in drug-resistant ovarian cancer was assessed in a subset of 109 patients with FIGO stage IIIc -IV disease and sub-optimal surgery. The resistant group was defined as presenting a first progression during or within 6 months following the end of treatment. The sensitive group was defined as non-progressive patients within the year after the end of treatment. *HER2* status between resistant and sensitive groups was evaluated using exact Fisher testing. *P* values ≤0.05 were considered significant. All analyses were performed using SPlus software (Insightful, Seattle, WA).

## Results

### *HER2* overexpression

**Figure 1:**
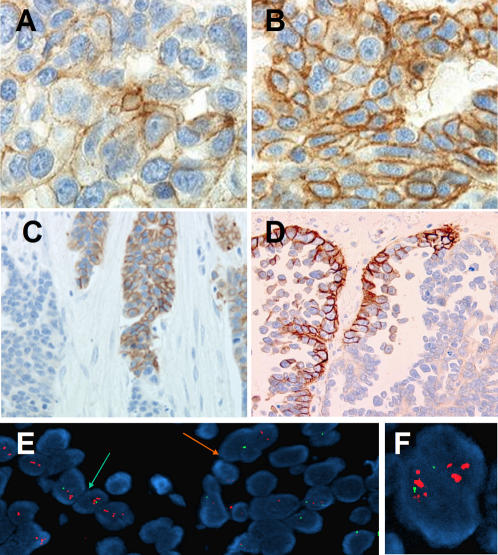
Immunohistochemistry labeling results. A. 2+ score: weak-to-moderate complete membrane staining in more than 10% of tumor cells (objX40). B. 3+ score: strong complete membrane staining in more than 10% of tumour cells (objX40). C. Heterogeneous staining of a primary ovarian tumour (objX20). D: Heterogeneous staining of a metastasis (objX20) E. FISH: heterogeneous amplification of *HER2* in a tumor showing a cluster of tumor cells with amplification (white arrow, left part) and a cluster of non amplified tumor cells (orange arrow, right part). F: Clusters of red spots (*HER2* amplification) together with two green spots (centromere 17).

Of the 320 tumors analyzed, *HER2* 3+ staining was observed in 15 samples (4.7%) and 2+ in 26 samples (8.1%) (Figure 1). *HER2* expression of 0 or 1+ was detected in the 279 remaining samples (87.2%). One-third of tumors showed some intracytoplasmic staining, considered as non-specific (data not shown). Among the 41 samples with 2+/3+ staining, 19 (46.3%) were heterogeneous and the same pattern was seen in the metastatic samples.

Of the 206 patients from whom both primary tumor and distant metastatic samples were available, there was concordance between primary tumor and metastases in 197 samples (95.6%; 179 negative and 18 positive). No statistical difference between *HER2* overexpression in primary tumors and corresponding metastases could be identified by kappa testing. In nine samples, 2+/3+ *HER2* staining was found exclusively in either primary tumor (five samples) or metastases (four samples). Three of these nine samples showed 3+ overexpression and were amplified. The six remaining samples showed 2+ expression and one of these showed *HER2* gene amplification by FISH.

### *HER2* gene amplification

**Figure 2:**
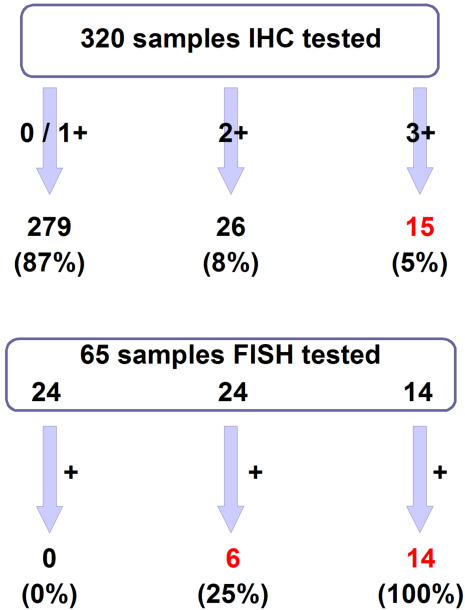
*HER2* gene amplification repartition according to *HER2* protein status. Perfect concordance in protein expression and gene amplification has been observed for samples with 3+ or 0/1+ IHC staining. In our study, 25% of equivocal samples (IHC 2+ staining) were amplified for *HER2* gene. Based on the HER2 reference scoring algorithm, 21 samples were considered as positive (15 scored 3+ by IHC and 6 scored 2+ validated by FISH). Three samples could not be compared by FISH due to fixation (one 3+ and two 2+ IHC scored). IHC = immunohistochemistry; FISH = fluorescence *in situ* hybridization.

Sixty-five samples were analyzed by FISH for *HER2* amplification, including all of the 26 equivocal (2+) tumors, the 15 positive (3+) tumors, and 24 samples showing 1+ or cytoplasmic staining. Three samples (two with 2+ and one with 3+ staining) were not evaluable because of DNA alteration by fixation, despite two attempts conducted in two laboratories. Thus a total of 62 samples were evaluated by FISH (Figure 2). In total 21 patients showed *HER2* positive status (all of the samples with IHC 3+ score and 6 of 24 samples with IHC 2+ score validated by FISH). None of the 24 samples with 1+ or cytoplasmic positivity was amplified for *HER2*.

In the heterogeneous samples, *HER2* overexpression and amplification were found in the same tumor areas. Eighteen of 21 samples with *HER2* amplification showed more than 8 copies per tumor nucleus with large clusters, suggesting homogeneous staining regions. The three remaining samples showed amplification with 8–10 copies per tumor nucleus, and a significant *HER2*/centromere 17 ratio.

### Relationship between biological markers and other prognostic variables

No relationship between *HER2* status and other prognostic factors (tumor stage, histological type, grade, ascites, debulking status, age and performance status) was found.

### Survival analysis

**Figure 3:**
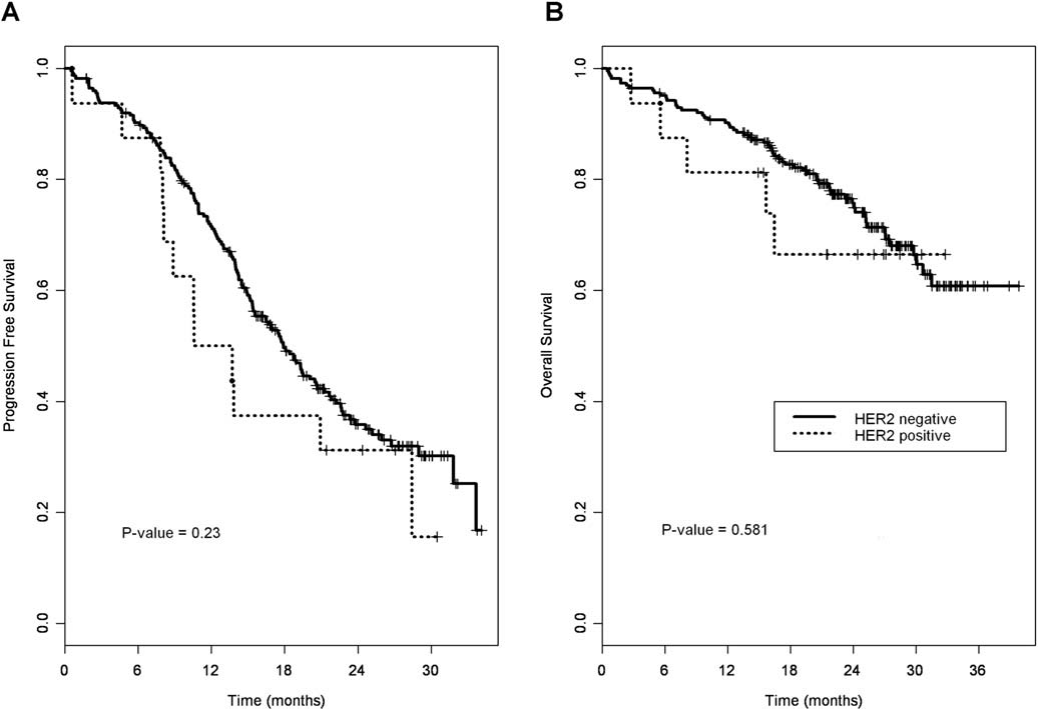
Progression-free survival and overall survival according to *HER2* status. A. Progression-free survival B. Overall survival

Median follow-up was 24.9 months (95% CI: 23.4–26.3). At the time of our analysis (July 2006), disease progression had occurred in 150 (61.7%) patients and 66 (27.2%) had died. Median PFS duration was 17.7 months (95% CI: 15.3–20.6). Median OS had not been reached. Among the 41 patients whose tumor was 2+/3+ by IHC, disease progressed in 18 (43.9%) and there were seven (17.1%) deaths, while in the group of 16 patients with *HER2* amplification, disease progressed in 12 (75%) and there were four (25%) deaths (Figure 3).

### Univariate analysis

Univariate analysis of the potential prognostic impact of clinical and histopathological parameters identified performance status 1 or 2, tumor stage, ascites and residual tumor after first laparotomy as significantly associated with shorter OS and PFS (Table 2). The association between age ≥ 60 years and poorer OS was borderline significant. *HER2* status (evaluated by either IHC or FISH) was not of prognostic value in terms of OS and PFS.

**Table 2:** Univariate analysis for progression-free survival and overall survival of biological and clinical parameters

Factor	Overall survival		Progression free survival	
	HR [95%CI]	P-value	HR [95%CI]	P-value
*HER2*	1.29 [0.37; 2.8]	0.58	1.4 [0.79; 2.59]	0.23
Positive vs. negative (IHC+FISH)				
*HER2*	0.809 [0.34; 1.87]	0.6	0.805 [0.49; 1.32]	0.39
IHC 2+/3+ vs 0/1+				
*HER2*	0.948 [0.51; 1.74]	0.86	0.809 [0.54; 1.19]	0.29
IHC 1+/2+/3+ vs 0				
Age	1.60 [0.97; 2.63]	0.057	1.22 [0.89; 1.7]	0.2
≥60 years vs <60 years				
Performance status	2.75 [1.54; 4.88]	0.00059*	1.62 [1.15; 2.26]	0.005*
1/2 vs 0				
Tumor stage	6.81 [1.66; 27.86]	0.0076*	4.37 [2.3; 8.38]	<0.00001*
III-VI vs I-II				
Ascites	3.07 [1.62; 5.78]	0.00048*	2.04 [1.41; 2.92]	0.00013*
Presence vs absence				
Residual tumour after first laparotomy	2.29 [1.33; 3.93]	0.0018*	2.3 [1.64; 3.22]	<0.00001*
>1 cm vs ≤1cm				

HR = Hazard ratio, CI = Confidence interval, * = P-value <0.05

### Multivariate analysis

Age, performance status, FIGO stage, ascites, residual tumor after first laparotomy and *HER2* amplification/overexpression status were considered. Only the presence of ascites was retained as an independent prognostic factor of both shorter PFS (*P* = 0.037) and OS (*P* = 0.016) (Table 3). High FIGO stage was also retained as a prognostic factor for PFS (*P* = 0.00041) alone. *HER2* status had no significant impact.

**Table 3:** Multivariate analysis of clinical parameters for progression-free survival and overall survival

Factor	Overall survival		Progression free survival	
	HR [95%CI]	P-value	HR [95%CI]	P-value
*HER2*	1.44 [0.51; 4.00]	0.49	1.67 [0.86; 3.22]	0.12000
Positive vs negative(IHC+FISH)				
Age	1.28 [0.73; 2.24]	0.380	1.06 [0.74; 1.52]	0.74
≥60 years vs <60 years				
Performance status	1.65 [0.86; 3.16]	0.12	1.26 [0.85; 1.85]	0.23000
1/2 vs 0				
Tumor stage	6.75 [0.17; 254.6]	0.064	3.96 [1.84; 8.4]	0.00041*
III-IV vs I-II				
Ascites	2.23 [0.79; 6,21]	0.016*	1.51 [1.02; 2.22]	0.037*
Presence vs absence				
Residual tumor after first laparotomy	1.16 [0.44; 3.03]	0.62	1.26 [0.84; 1.86]	0.26
>1 cm vs ≤1 cm				

HR = Hazard ratio, CI = Confidence interval, * = P-value <0.05

### Chemoresistance and *HER2* status

From the cohort of patients included in the TCG trial, a subset of 109 patients with FIGO stage IIIc/IV primary tumor and sub-optimal surgery was selected for analysis. Based on follow-up, 46 patients were considered as chemoresistant, 36 as chemosensitive, and 27 could not be classified in one of the two groups. *HER2* status was not significantly linked to chemoresistant status.

## Discussion

**Figure 4:**
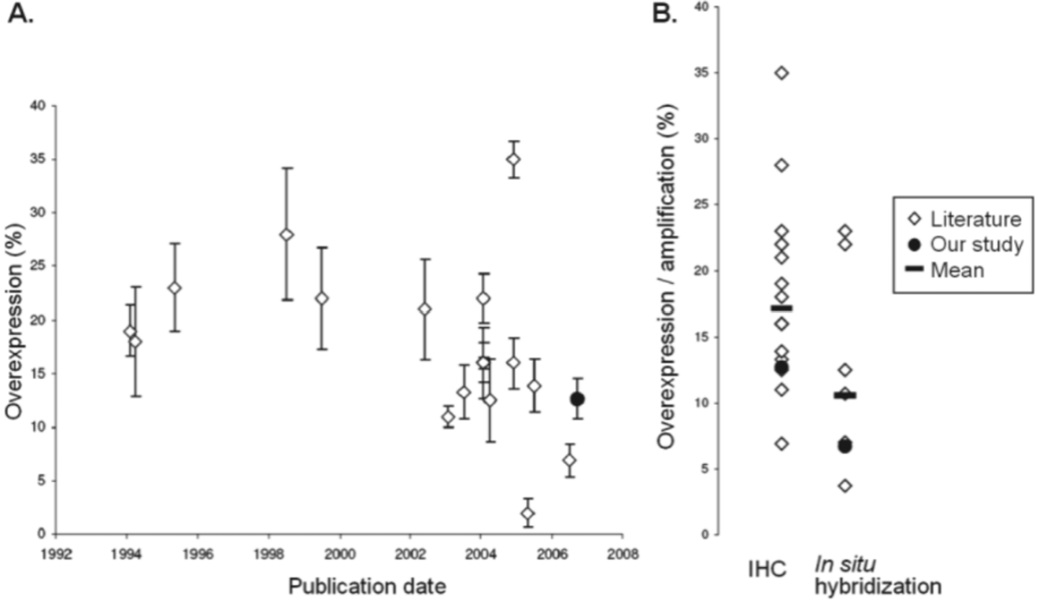
*HER2* in major published studies. A. Overexpression Review of selected articles evaluating *HER2* protein expression in large series of patients (including more than 50 tumour samples) published in international journals after 1994. Boxes represent % of *HER2* overexpression (scored as 2+ or 3+) and error bars show ±2 standard errors for each study. B. IHC and FISH status Review of selected articles evaluating *HER2* gene amplification (FISH or CISH) and/or *HER2* protein expression in large series of patients (including more than 50 tumour samples) published in international journals after 1994.*In situ* hybridisation represents FISH (fluorescence *in situ* hybridisation) and CISH (chromogenic *In situ* hybridisation) results. Mean *HER2* overexpression/amplification across studies is represented; IHC = Immunohistochemistry.

In this study, we screened 320 advanced ovarian cancers for *HER2* status. To our knowledge, this is the first large multicentric study investigating both primary tumor and metastases on conventional slides by IHC and FISH techniques. Positive *HER2* status was found in 6.6% (21 of 320) of the tumor samples. The rate of *HER2* positivity varies in the literature from 8% to 66% [15]–[33] (Figure 4). There are several possible explanations for the wide variation and the relatively low rate of *HER2* positivity reported in our series, including the different detection methods used (IHC, FISH and chromogenic *in situ* hybridization), different sources of material (blocks of tumors and tissue microarray), and variations in IHC assay techniques (CB-11, HercepTest or a non-commercial antibody); in addition, variance in staining protocols and subjective interpretation of sample stains makes direct comparison of studies difficult. The present study has the advantage of being based on a large, prospective, multicenter trial, with extensive tumor sampling (four conventional slides of tumor/metastases when available). Moreover, all of the positive/doubtful samples (IHC 1+, 2+, 3+ and those with cytoplasmic staining) were analyzed by FISH. The rate of *HER2* protein overexpression (2+/3+ by IHC) was 13% in the present series, whereas it varied from 1.9% to 35% in previous reports. We used the CB-11 monoclonal antibody, which has been shown to be more accurate than HercepTest, the other widely used, Food and Drug Administration (FDA)-approved antibody [12], [34], and to show better concordance with FISH ([11], [35]). To our knowledge, no cross-reactivity with other EGFR family protein has been reported using this antibody [31]. We adapted our IHC assay according to Couturier et al. [36] with a dilution of the primary antibody (1/800), since this procedure has shown a complete concordance with gene amplification assessed by FISH. Our results confirm the good concordance between IHC and FISH analyses in ovarian cancers because all the samples showing 1+ staining or cytoplasmic staining were not amplified, thus excluding the hypothesis of possible false negatives. It is worth noting that *HER2* testing procedures have evolved with time, resulting in a more consistent rate of positive cases (11–16%) in most of the recent studies.

Compared with the 20–30% rate of *HER2* positivity observed in breast cancers [34], [37], the rate in ovarian cancer is lower and intratumoral heterogeneity is frequently detected. The good concordance between *HER2* status in primary tumor and corresponding distant locations suggests that *HER2* clonal selection occurs before tumor dissemination. It is worth noting that some tumors showed the same heterogeneous pattern in both primary and distant locations with adjacent positive and negative areas, detected by both IHC and FISH methods.

We observed a good correlation between IHC and FISH results for no/weak (0/1+) and strong (3+) IHC scores, whereas only 25% of the IHC 2+ samples were found to be amplified by FISH testing. A similar rate has been reported in breast cancer [10], [38]. Moreover, it has been shown that among 2+ IHC false positive cases, none overexpressed HER2 mRNA as mesured by in situ hybridization [12]. Our results show that the decision algorithm currently used in breast cancers (IHC as a screening test, with equivocal results confirmed by FISH) is appropriate in ovarian cancers. *HER2* amplification/overexpression is associated with poor prognosis in several cancer types, and its prognostic value in ovarian cancer has been reviewed recently by Serrano-Olvera et al. [39]. An adverse prognostic impact of *HER2* overexpression and/or amplification has been shown in most of the published series [3], [16]–[18], [23], [27], [28], [30], [32], [40], including one reported by our group [24]. In the present study, we did not identify any prognostic value of *HER2* status. In contrast to our previous study [24], patients in the present series received paclitaxel-based chemotherapy, raising the question of a possible interaction between *HER2* positivity and drug sensitivity. The clinical impact of *HER2* status on the response to paclitaxel has been suggested, but a significant association has not been shown [41]–[42]. Interestingly, *in vitro* studies in the SK-OV-3 ovarian cancer cell line showed that sensitivity to paclitaxel dramatically increased in cells expressing high levels of *HER2* [43]. Since HER2-positive cells are likely dividing rapidly, they may be more sensitive to paclitaxel-based chemotherapy. Altogether, these results suggest that paclitaxel may overcome the poor prognosis associated with *HER2* positivity targeting dividing cells.
